# Non-Hodgkin’s Lymphoma Presenting as Constrictive Pericarditis: A Rare Case Report

**Published:** 2016-04-13

**Authors:** Maryam Nabati, Keyvan Yosofnezhad, Morteza Taghavi, Ali Abbasi, Ali Ghaemian

**Affiliations:** 1 *Department of Cardiology, Faculty of Medicine, Mazandaran University of Medical Sciences, Sari, Iran.*; 2 *Student Research Committee, Faculty of Medicine, Mazandaran University of Medical Sciences, Sari, Iran.*; 3 *Department of Pathology, Faculty of Medicine, Mazandaran University of Medical Sciences, Sari, Iran.*

**Keywords:** *Pericarditis- constrictive*, *Lymphoma- non- Hodgkin*, *Diagnosis*

## Abstract

Constrictive pericarditis (CP) is an uncommon post inflammatory disorder. It is described as pericardial thickening, myocardial constriction, and impaired diastolic filling. The most common etiologies are idiopathy, mediastinal radiotherapy, and prior cardiac surgery. Less common etiologies include viral infections, collagen vascular disorders, renal failure, sarcoidosis, tuberculosis, and blunt chest trauma. CP can less commonly be caused by malignancy. We report a very rare case of non-Hodgkin’s lymphoma (NHL) presenting twice with attacks of decompensated heart failure. Echocardiography revealed that CP was responsible for the patient's symptoms as the first manifestation of NHL. Chest computed tomography scan and biopsy findings were compatible with the diagnosis of NHL. The patient received R-CHOP (cyclophosphamide, hydroxydaunorubicin, Oncovin^®^, and prednisone or prednisolone, combined with the monoclonal antibody rituximab) chemotherapy. Three months later, there was significant improvement in the patient’s symptoms and considerable decrease in pericardial thickness.

## Introduction

Constrictive pericarditis (CP) is an uncommon post-inflammatory disorder. It is described by pericardial thickening, myocardial constriction, and impaired diastolic filling. Small effusions are usually detected between adhesions.^1^ The most common etiologies are idiopathy, mediastinal radiotherapy, and prior cardiac surgery. Less common etiologies include viral infections, collagen vascular disorders, renal failure, sarcoidosis, tuberculosis, and blunt chest trauma.^1^^, ^^2^ CP can less commonly be caused by malignancy.^2^

The existing literature contains only a few case reports on CP caused by the infiltration of non- Hodgkin’s lymphoma (NHL)^3^ and no case reports characterizing CP as the first manifestation of NHL.

Here we report a very rare case of NHL presenting twice with attacks of decompensated heart failure. Echocardiography revealed that CP was responsible for the patient's symptoms of heart failure as the first manifestation of NHL.

## Case Report

A 62-year-old woman was admitted to our hospital with respiratory distress and cyanosis, complaining of orthopnea and paroxysmal nocturnal dyspnea. There was no fever or sweating during her hospitalization. In her past medical history, there was not any known cardiovascular risk factor. She had a previous admission to our hospital because of decompensated heart failure, which had occurred 7 months previously. Thereafter, she was relatively well, but had experienced gradual worsening of dyspnea recently.

**Figure 1:**
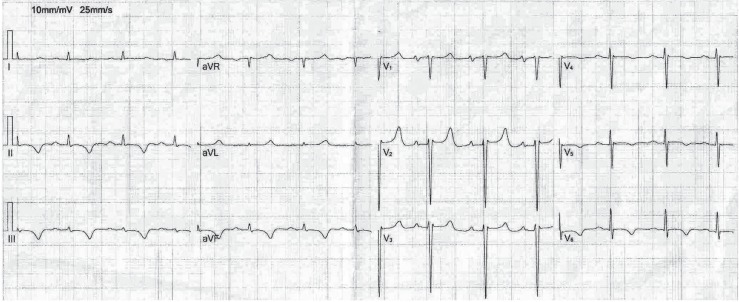
Electrocardiogram shows sinus tachycardia, normal axis, and T-wave inversion in leads II, III, aVF, V5, and V6.

**Figure 2:**
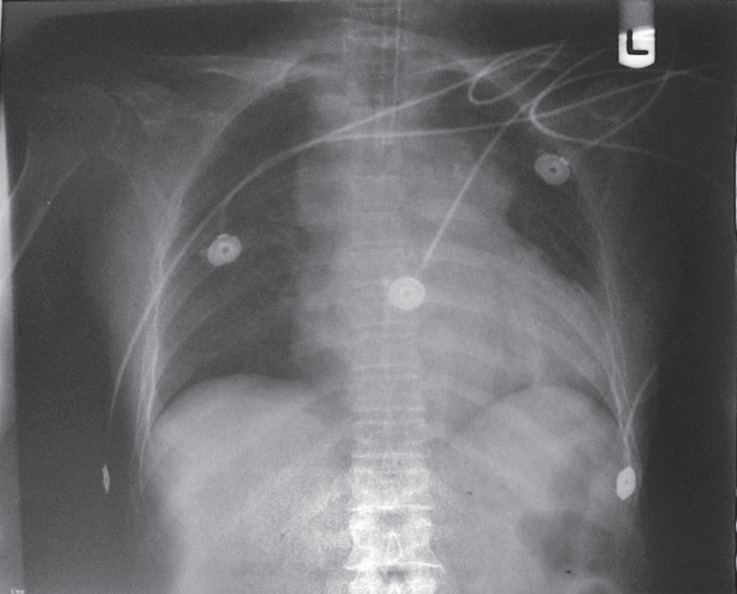
Posteroanterior chest X-ray illustrates cardiomegaly, pulmonary congestion, and widened superior mediastinum.

Upon physical examination, there was jugular venous distension and peripheral cyanosis. An electrocardiogram (Cardisuny C 120, Fukuda M-E Kogyo Co., Ltd., Tokyo, Japan) showed sinus tachycardia, normal axis, and T-wave inversion in leads II, III, aVF, and V5-6 (Figure 1). Posteroanterior view of chest radiography illustrated cardiomegaly, pulmonary congestion, and widened superior mediastinum (Figure 2). Transthoracic echocardiography (TTE), followed by transesophageal echocardiography (TEE), demonstrated normal left ventricular size and systolic function, normal right ventricular size and systolic function, mild mitral regurgitation, mild tricuspid regurgitation, and high normal pulmonary artery pressure (35 mm Hg). The inferior vena cava was dilated with blunting of respiratory collapse. Also, there was inspiratory diastolic flow reversal in the hepatic vein. Two-dimensional echocardiography images revealed ventricular septal bouncing.

**Figure 3:**
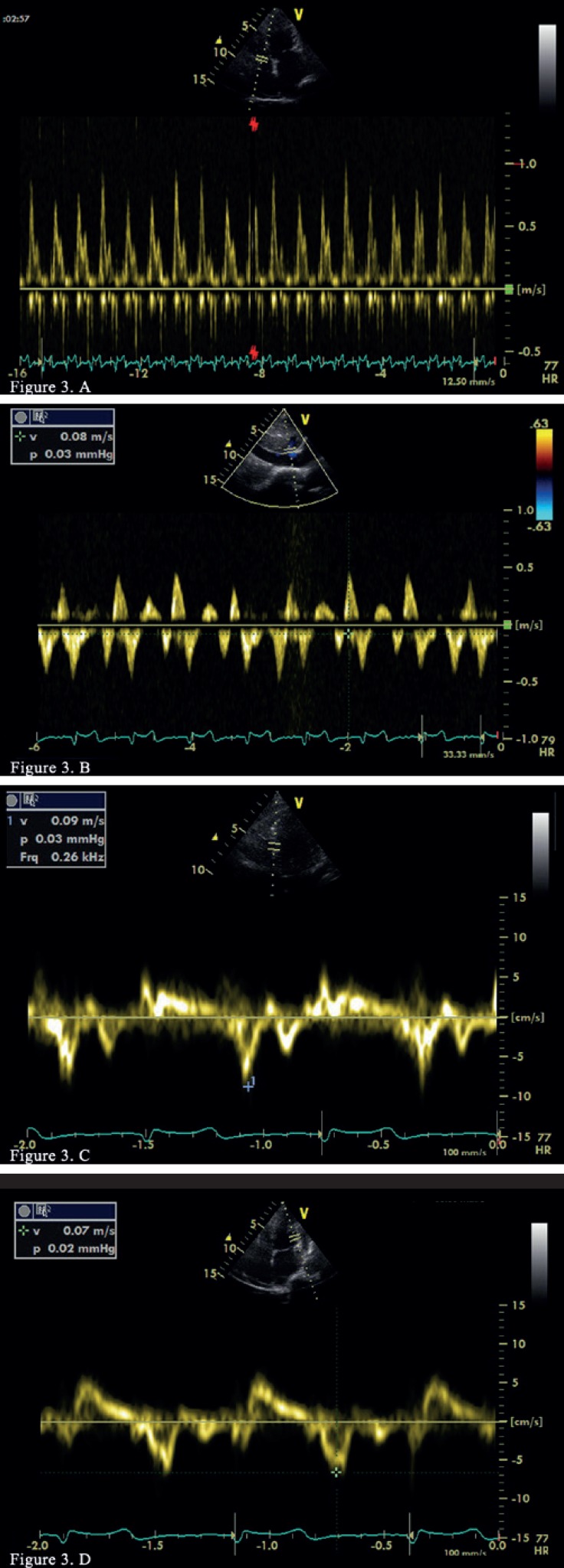
Pulsed-wave Doppler echocardiography demonstrates a restrictive filling pattern and significant respiratory variation in the tricuspid valve inflow (A) and inspiratory diastolic flow reversal in the hepatic vein (B). Tissue Doppler echocardiography reveals annulus reversus (C and D; septal and lateral tissue E' velocity, respectively).

In Doppler imaging, there was a restrictive filling pattern in the mitral and tricuspid inflow (high peak early diastolic velocity [E wave]), a short deceleration time, and a low peak late diastolic velocity (A wave) and significant respiratory variation. Tissue Doppler imaging showed septal E' velocity (tissue Doppler mitral annular early diastolic velocity) of 9 cm/s and annulus reversus (septal E' was larger than lateral E') (Figure 3). TEE-recorded pulmonary vein flow revealed a lower systolic-to-diastolic filling ratio.

**Figure 4:**
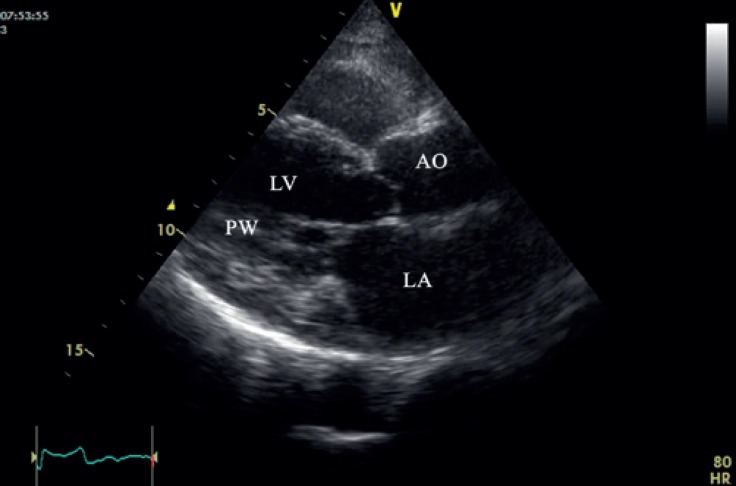
Transthoracic 2-dimensional echocardiography in the parasternal long-axis view reveals a severely thickened pericardium (arrow).

AO, Aorta; LA, Left atrium; LV, Left ventricle; PW, Posterior wall

**Figure 5:**
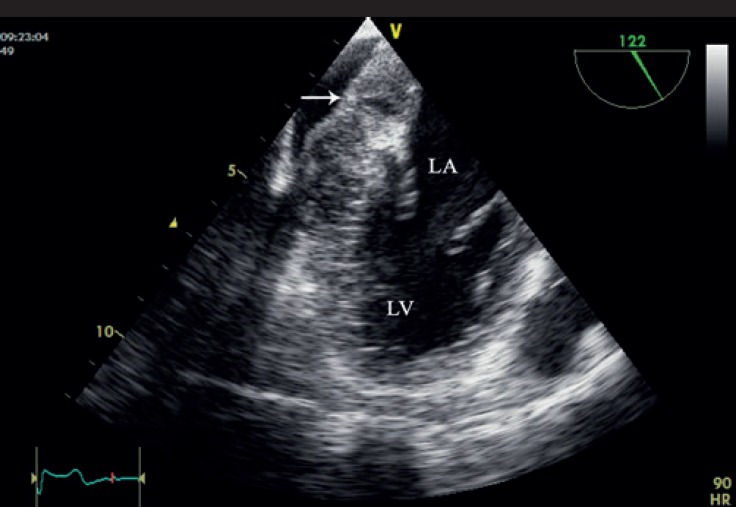
Transesophageal 2-dimensional echocardiography in the mid-esophageal view demonstrates a severely thickened pericardium (arrow).

LA, Left atrium; LV, Left ventricle

**Figure 6:**
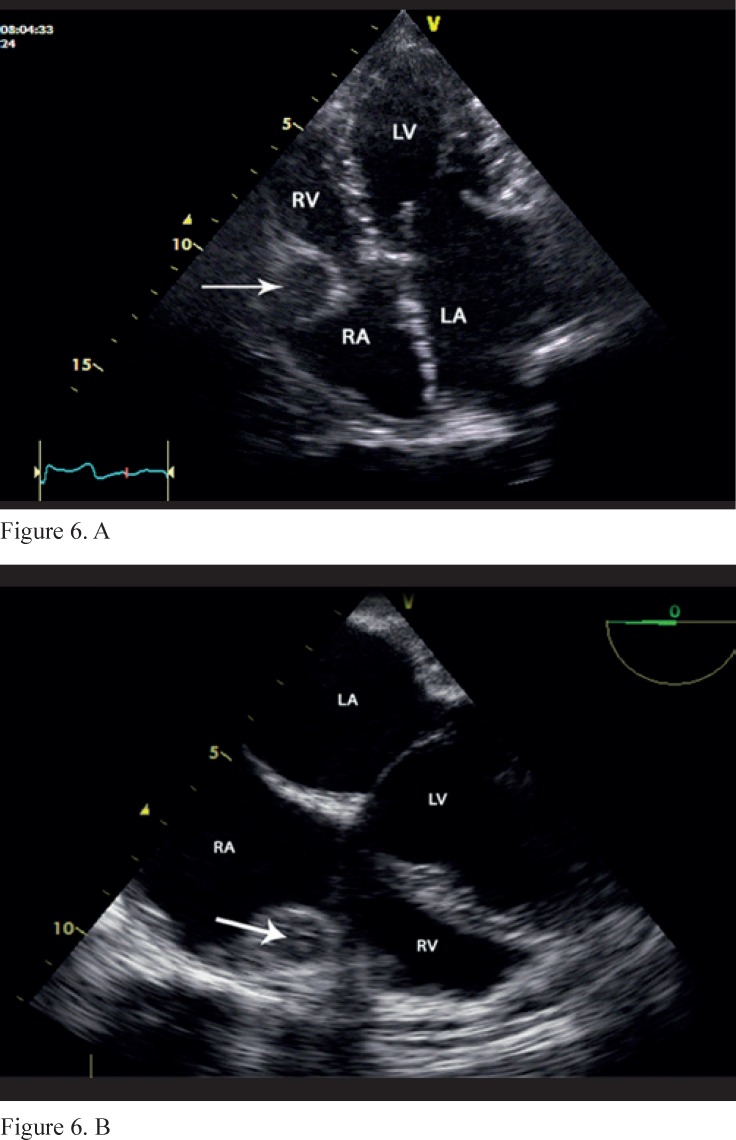
Transthoracic (A) and transesophageal (B) 2-dimensional 4-chamber view shows mass-like lesions (arrow) in the right atrioventricular groove region which partially obstruct the tricuspid valve inflow.

LA, Left atrium; LV, Left ventricle; RA, Right atrium; RV, Right ventricle

In 2-dimensional and M-mode echocardiography, the pericardium was severely thick (> 1 cm) (Figure 4 and Figure 5): it appeared as mass-like lesions in the atrioventricular groove and partially obstructed the tricuspid valve inflow (7.9 and 3.8 mm Hg, peak and mean pressure gradients across the tricuspid valve, respectively) (Figure 6). These data altogether were indicative of CP.

**Figure 7:**
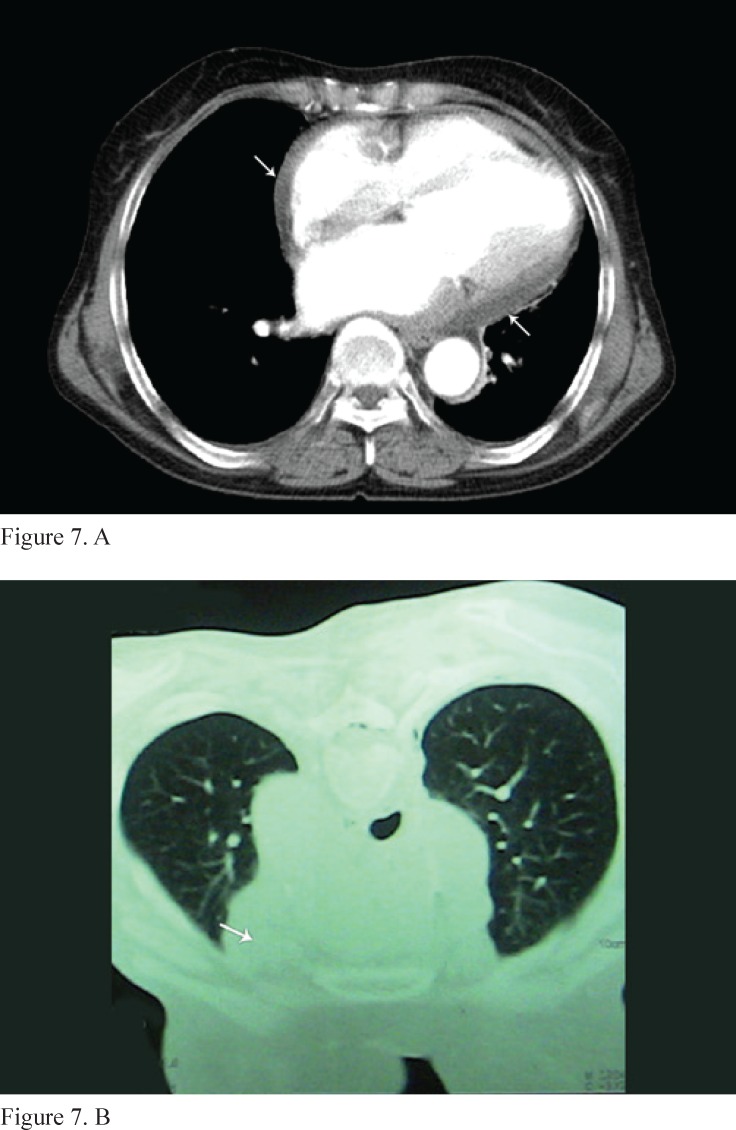
Chest computed tomography scan, without (A) and with (B) contrast, shows a severely thickened pericardium (arrow).

Subsequently, the etiology was determined via chest and abdominal computed tomography (CT) scan (Siemens Medical Solutions, Forchheim, Germany). Chest CT illustrated multiple swollen lymph nodes in the prevascular regions of the anterosuperior mediastinum and the aortopulmonary window. Also, there was a large mediastinal mass, which encrusted the heart (Figure 7). Abdominal CT scan showed no abdominal involvement.

Thereafter, single-photon emission computed tomography (SPECT) revealed normal coronary arteries.

Complete blood count showed mild anemia (a hemoglobin level of 11.4 mg/dL), but white blood cell and platelet counts were in the normal range. There was a mild elevation in aspartate aminotransferase level. However, blood chemistry did not show any other abnormality.

**Figure 8:**
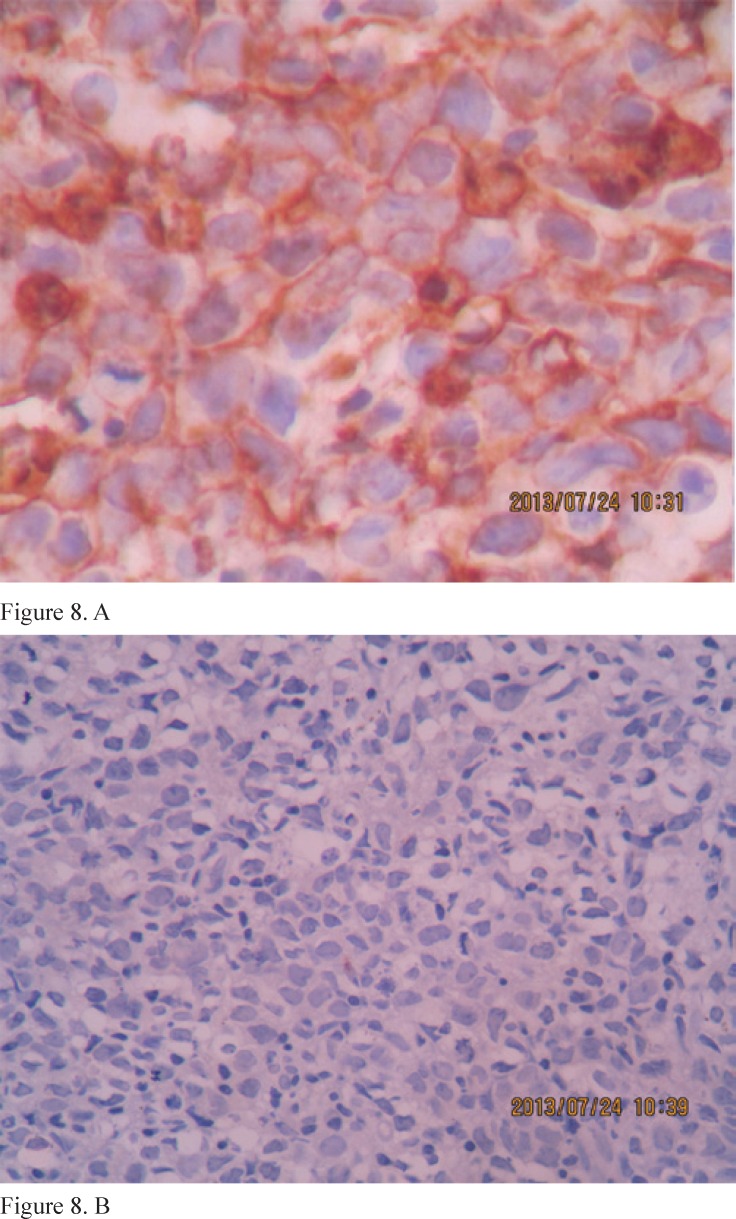
Immunohistochemical examination demonstrates a positive reaction to CD20 antigen (A) and a negative reaction to cytokeratin (B).

A biopsy from the para-thoracic lymphoid mass revealed NHL (diffuse large B cell lymphoma, whereby the lymph nodes were infiltrated by large lymphoid cells with positive CD20, positive CD45, and CD79 and negative CD5, CD10, CD15, CD23, and CD30 as well as negative cytokeratin) (Figure 8).

The biopsy is taken from the para-thoracic lymphoid mass.

The patient received R-CHOP chemotherapy (CHOP consisted of cyclophosphamide, hydroxydaunorubicin, Oncovin^®^, and prednisone or prednisolone, combined with the monoclonal antibody rituximab). After three courses of chemotherapy, TTE showed a significant decrease in the pericardial thickness. Furthermore, the M-mode, Doppler, and tissue Doppler signs of CP had disappeared.

## Discussion

The echocardiographic diagnosis of CP was originally based on M-mode echocardiographic findings and subsequently on 2-dimensional echocardiography and Doppler hemodynamics in response to the respiratory cycle. More recently, newer echocardiographic techniques, such as pulsed tissue Doppler, color Doppler tissue imaging (DTI), and speckle-tracking imaging, have been used to assess the unique changes in the global and regional myocardial function seen in CP.^4^ Mitral inflow, as assessed by Doppler echocardiography, demonstrates an increased early diastolic filling velocity, followed by rapid deceleration, leading to a short filling period.^5^

Dynamic changes with respiration occur in patients with CP. Typically, patients with CP demonstrate an increase in early diastolic mitral inflow velocity of ≥ 25% during expiration compared with inspiration.^6^ Pulsed Doppler recordings of the hepatic vein flow in CP show marked diastolic flow reversal, which increases in expiration compared with inspiration.^7^ Doppler evaluation of the pulmonary veins demonstrates a marked respiratory change in the pulmonary venous flow in CP. The pulmonary venous systolic wave and early diastolic wave velocities, especially the early diastolic wave velocity, are increased during expiration and decreased during inspiration. Color M-mode Doppler illustrates early-onset, elevated mitral inflow velocities in patients with CP.^4^

Abrupt anterior or posterior motion of the interventricular septum in early diastole is common in patients with CP. In classic CP, the interventricular septum shows a brisk, early diastolic motion toward the left ventricle during inspiration, followed by a rebound in the opposite direction during expiration. This septal bounce reflects exaggerated interventricular dependence combined with forceful early diastolic filling.^8^

In patients with CP, mechanoelastic properties of the myocardium are relatively preserved in the longitudinal direction and, therefore, longitudinal deformation of the left ventricular base and longitudinal early diastolic velocities are either normal or exaggerated. A lateral or septal early diastolic mitral annular velocity of > 8 cm/s on pulsed tissue Doppler is in general the accepted cut-off value to distinguish patients with CP from those with restrictive cardiomyopathy.

In CP, the lateral E' velocity is lower than the medial E' velocity, resulting in annulus reversus.^9^ Patients present predominantly with heart failure with elevated jugular venous pressure, dyspnea, peripheral edema, hepatomegaly, and ascites.^4^ CP can less commonly be caused by malignancy.^2^ Malignancy can manifest as pericardial effusion (with or without tamponade) or may encrust the heart with thickening of the pericardial layers, resulting in a constrictive physiology.^2^ Secondary involvement of the heart is much more frequent than primary tumors. Lung and breast cancers are the most frequent causes of malignant pericardial disease. However, lymphoma can also involve the pericardium.^10^ In our patient, para-aortic lymph node involvement revealed that cardiac involvement had been created by secondary invasion. The most frequent secondary malignant tumor which involves the heart is adenocarcinoma.^11^

Cardiac involvement may develop as a result of lymphatic, hematogenous, or direct extension. Lymphatic spread is the most frequent pathway, and it usually involves the pericardium.^2^

NHLs are a heterogeneous group of lymphoproliferative malignancies. Predominant involvement of the heart by NHLs is usually detected post mortem. They are usually the immediate cause of death.^12^ Types of NHLs vary significantly in their severity, from indolent to very aggressive.^13^

The existing literature contains only a few case reports on CP caused by the infiltration of NHL3 and no case reports characterizing CP as the first manifestation of NHL.

CP by diffuse pericardial thickening of a metastatic origin is a rare complication, and it would be very unusual for such constriction to be the first manifestation of a neoplastic process.^2^

In our patient, CP was the primary presentation of diffuse large B-cell lymphoma. Diffuse large B-cell lymphoma can present with nodal or extra nodal disease. It is in differential diagnosis with primary mediastinal large B-cell lymphoma. However, the latter is limited to the mediastinum. These patients usually present with a single, rapidly enlarging mass which may be disseminated. It is seen more frequently between 20 and 40 years. In spite of diffuse large B-cell lymphoma, lymphoma cells are commonly CD23 positive.^14^

## Conclusion

Malignancies must be considered in the differential diagnosis of any patient presenting with CP.

## Notes:

***This paper should be cited as:*** Nabati M, Yosofnesuad K, Taghavi M, Abbasi A, Ghaemian A. Non-Hodgkin’s Lymphoma Presenting as Constrictive Pericarditis: A Rare Case Report. J Teh Univ Heart Ctr 2016;11(2):92-97.

## Supplementary Materials

The following supplementary material is available online:

Movie clips for Figures 4–6:

Video clip4.avi, Video clip5.avi, Video clip6a.avi, and Video clip6b.avi

http://jthc.tums.ac.ir/index.php/jthc/article/view/586/460

http://jthc.tums.ac.ir/index.php/jthc/article/view/586/461

http://jthc.tums.ac.ir/index.php/jthc/article/view/586/462

http://jthc.tums.ac.ir/index.php/jthc/article/view/586/463

The file names of the movie clips correspond to the figure numbers.

Supplementary Data: Video clip4 Video clip5 Video clip6a Video clip6b
